# Systematic review: the impact of policy levers on mental health service utilization and access for Autistic children

**DOI:** 10.1186/s13034-025-00963-9

**Published:** 2025-10-08

**Authors:** Sara Cibralic, Lulu Barker, Patrick Hawker, Bruce Tonge, Katrina Williams, Elizabeth J Elliott, Mark Bellgrove, Tim Silk, Vicki Anderson, Michael Kohn, Emma Sciberras, Valsamma Eapen

**Affiliations:** 1 School of Clinical Medicine, University of New South Wales, Sydney, NSW Australia; 2 Academic Unit of Child Psychiatry, South Western Sydney Local Health District, Sydney, NSW Australia; 3 Monash University, Melbourne, VIC Australia; 4 University of Sydney, Sydney, NSW Australia; 5 The Children’s Hospital at Westmead, Sydney, NSW Australia; 6 Deakin University, Melbourne, VIC Australia; 7 Murdoch Children’s Research Institute, Melbourne, VIC Australia; 8 University of Melbourne, Melbourne, VIC Australia; 9 Western Sydney University, Campbelltown, NSW Australia; 10 Campbelltown Hospital, Campbelltown, NSW Australia; 11 Alfred Infant, Child and Youth Mental Health and Wellbeing Service, Melbourne, VIC Australia

**Keywords:** Autism spectrum disorder, Mental health services, Health policy, Health care accessibility, Systematic review

## Abstract

**Objective:**

Autistic children’s ability to access mental health services can be challenging due to the limited availability of therapists with autism experience, service ineligibility, and financial strain. This systematic review evaluated and synthesized literature regarding the impact of government policy levers on the access to, and utilization of, mental health services by Autistic children and their families.

**Method:**

Interdisciplinary databases together with gray literature and supplementary searches were used to identify relevant articles. Peer-reviewed, English language studies which reported on the impact of government policy levers on the utilization of, and access to, mental health services by Autistic children and their families were included.

**Results:**

Searches resulted in the identification of 2305 articles (database searches = 744, additional searches = 1531), six of which were included in the final review. All six articles were from the United States of America, published between 2013 and 2020, with a focus on national and state regulatory policy levers targeting insurance companies. Results indicated that most policy levers did not improve service access to, or utilization of, mental health services. Gray literature searches identified that several countries had implemented autism specific policy levers, most however had not been evaluated regarding their impact on mental health service access and utilization by Autistic children or their families.

**Conclusion:**

The majority of identified policy levers have not resulted in greater utilization or access of mental health services for Autistic children or their families. More global research, focusing on datasets that have allowed policies time to impact change, is needed.

## Introduction

Autism Spectrum Disorder (hereafter referred to as autism) is a neurodevelopmental condition characterized by impairments in social communication and interaction as well as restricted, repetitive behaviors, interests, and/or activities [1]. The global prevalence of autism is estimated to be approximately 1% [2], with much higher rates, one in 36 children aged 8-years, reported in the USA [3]. The differences in incidence rates of autism have been attributed to several potential factors, including changes in diagnostic practice as well as service access [4]. Current evidence indicates that Autistic children are at a higher risk of having co-occurring mental health conditions, with greater than 70% of children experiencing at least one psychiatric disorder [5–7].

### Autism and access to mental health services

The immediate and long-term consequences of having a co-occurring mental health condition for Autistic children and their families are well known [8–11]. Despite this, frequent reports indicate that Autistic children with co-occurring psychiatric conditions have unmet service needs [12, 13], with unexperienced therapists, ineligibility for specific services, financial strain, funding for sessions/insurance coverage, and location of services [14–16] being frequently cited as barriers to service access and utilization. A recent systematic review [15] of 12 studies (all undertaken in WEIRD (Western, Educated, Industrialized, Rich, and Democratic) countries using surveys, interviews, or focus groups to collect data), for example, found that the most frequently cited barrier to accessing mental health services by both Autistic children and adults was a therapists’ lack of, or limited, knowledge and/or experience of autism together with services unwilling to alter treatments to fit the needs of Autistic people. Other commonly cited barriers pertaining to Autistic children included funding/insurance coverage, not meeting mental health service entry criteria, and being referred between mental health and disability services. This disparity has been shown to be even more evident in Autistic children from racial and ethnic minority groups and lower socioeconomic status [17]. For example, a systematic review [17] of 11 United States of America (USA) studies, which were either cross-sectional studies (*n* = 2; 1 = classroom and home settings and 1 = home setting), qualitative studies (*n* = 1, clinic and home setting), or secondary analysis of national (*n* = 5), medical (*n* = 1), or community survey data bases (*n* = 1) (setting not reported), showed that Autistic children from Hispanic and African backgrounds were less likely to use specialist services, including psychiatry and psychology. The obstacles to service access included language barriers, not having a personal doctor, and difficulty acquiring referrals. The review also found that Autistic children from lower socioeconomic status backgrounds had less access to, and use of health services compared to children from higher socioeconomic status backgrounds. Barriers to access were largely attributed to difficulties receiving a referral to specialist services. In terms of service utilization, lower levels of parent education were associated with lower levels of service use.

### Policy levers

Governments, through the implementation of policy levers (i.e., tools or instruments through which governments can attain goals), can improve access to, and utilization of, services [18]. Five types of health sector policy levers have been outlined by Roberts, Hsiao [19].The first, *Financing*, refers to the mechanisms (e.g., taxes) for raising funds to pay for health sector activities. The available funding impacts the types of treatment options that are reimbursed including autism specific services. The second, *Payment*, relates to the methods of transferring money to health care providers (e.g., capitation). The way that providers are reimbursed can influence their desire to work within a system. If insurance companies, for example, delay payments pertaining to work undertaken with Autistic individuals, providers may choose to stop offering these services. Third, *Organization*, indicates the structure of the organization (e.g., private hospitals) as well as individual institutes providing health care services (e.g., sole practitioners). Organizational capacity to provide language services, for example, can impact access for Autistic children from racial and ethnic minorities [17]. Fourth, *Regulation*, is concerned with the use of legislation to guide or influence behavior of those within the health system (e.g., insurance company coverage of services for Autistic individuals [15]). Fifth and final, *Behavior*, deals with attempts to influence how individuals act relating to their health and health care (e.g., media health campaigns to encourage Autistic individuals to sign up to insurance coverage tailored to their needs).

Policies can have elements of one or all types of the above-mentioned levers. For example, in the USA, The Paul Wellstone and Pete Domenici Mental Health Parity and Addiction Equity Act (MHPAEA) of 2008 (also known as the federal parity law) is a regulatory lever introduced to ensure equal benefits for mental/behavioral health and medical/surgical needs [20]. The Home and Community-Based Services 1915(c) waiver [21], another USA policy lever, has elements of financing and organizational levers as it allows for Medicaid funds to be allocated towards non-institutional care options and also helps structure the delivery of home and community-based services through the development of eligibility criteria. The Australian National Disability Insurance Scheme Act 2013 [22, 23], a personalized funding model, is an example of a primarily financing lever as it provides a structured mechanism for raising and allocating funds relating to the health and social care systems. It also has elements of organization and regulation levers. It oversees service delivery, and, in the National Disability Insurance Scheme Act 2013, sets out standards, eligibility criteria, and compliance requirements for providers and participants, which influences behavior and accountability within the system.

While these policy levers have been implemented to improve service accessibility and have, for the most part, been found to be delivering the outcomes they were designed to deliver [24, 25], they have not been without criticism. Research has shown that while enrollments in The Home and Community-Based Services 1915(c) waiver program, for example, have steadily increased, there have been inequalities regarding who has enrolled in the program [24]. A study by Levine, Cole [24] , which analyzed 2016–2019 Medicaid data of adults with Down syndrome, autism, and intellectual disability, found that individuals with Down syndrome were more likely on average to be enrolled in the program while Autistic adults were least likely on average to be enrolled in the program. The authors noted that this inequality may be influenced by how individuals experience administrative tasks related to documenting ‘visible’ (i.e., Down syndrome) versus ‘invisible’ (i.e., autism) disabilities. Similarly, the NDIS has been criticized for its potential to exacerbate inequality in service access [25, 26]. A recent literature review [26], which identified 11 studies focusing on the NDIS and equitable access, found that the structure of the NDIS administrative system favors those who can understand and take on the administrative and decision-making tasks. In doing so, it has the potential to enhance existing inequalities in service accessibility. Consequently, specific groups of people with disabilities, including those with psychosocial disabilities, have reported struggling with the NDIS administrative process [25].

### Policy levers and mental health service access

Previous empirical studies of policy levers targeted at mental health service access and utilization have shown that they can be effective at reducing barriers to obtaining mental health care by children and their families [27, 28]. A systematic review [27] of 20 studies undertaken in the USA, for example, found that specific policy levers such as location-based policy levers (e.g., integrated care models) and insurance-based levers (e.g., public insurance) were associated with higher utilization and acceptability of services as well as affordability, respectively. However, less is known about the impact of such policy levers on Autistic children and their families, with the So, McCord [27] review only identifying one study focused on autism. The sole study [29] identified by So, McCord [27] indicated that a policy targeted at improving health insurance cover for those with autism did not have an impact on families’ perceived financial burden. The authors noted potential issues with the study design (i.e., insufficient time between mandate and expected changes in affordability) as a possible reason for the finding. There are several possible reasons as to why more studies focusing on Autistic children were not identified in So, McCord [27] review, including that the research focused on USA based studies, the research topic may not have been a government research priority area in the USA or internationally [30], or because historically Autistic children have often been excluded from mainstream research [31].

### Current study

Given that the review by So, McCord [27] was focused on USA studies only and undertaken in 2017 (published in 2019), research conducted in other countries may be available and new USA research data could have emerged. This review therefore aimed to examine all available empirical literature with a view to narratively synthesize the impact of policy levers on the access to, and utilization of, mental health services by Autistic children (0–17 years and 11 months) and their families. The following research questions were addressed:

1. What government policy levers have been implemented to improve mental health service access and utilization among Autistic children, and how have they been evaluated?
2. What are the impacts of these policy levers on mental health service utilization and access by Autistic children and their families?

## Method

This systematic review was conducted following the Preferred Reporting Items for Systematic Reviews and Meta-Analyses (PRISMA) guidelines [32]. Before commencement, the review protocol was registered with the University of York Centre for Reviews and Dissemination (PROSPERO; registration number CRD42024609816).

### Search strategy

Six search strategies were implemented to identify relevant literature. First, databases (PsycINFO, EMBASE, PubMed, SCOPUS and CINAHL) were searched concurrently for peer-reviewed articles published from inception to October 2024. The following broad search terms, including their synonyms and medical subject headings (MeSH terms) when available, were methodically entered into each database (see Table 1 for an overview and Supplementary Table 1 for the exact search terms used in each database):

**Table 1:** Search terms

#	Field(s)	Search term(s)—applied to title (TI) OR abstract (AB) fields only
1	TI OR AB	autis* OR neurodevelop* OR asperg*
2	TI OR AB	“mental health” OR “mental disorder*” OR mental illness* OR psychosocial OR behavio*
3	TI OR AB	child* OR adolescen* AND treat* OR therap* OR training OR service* OR care OR healthcare
4	TI OR AB	polic* OR law OR statut* OR regulat* OR reform OR legislat* OR bill OR rule* OR NDIA OR NDIS OR insurance
5	TI OR AB	access* OR availab* OR afford* OR cost* OR coverage OR deliver* OR barrier* OR pay OR “pay for performance” utilization
6		1 AND 2 AND 3 AND 4 AND 5
7		Limit to age = 0–18 years
8		Limit to English Language
9		Limit to human

Second, the ‘cited by’ and ‘related articles’ options available on Google Scholar were used to manually search for articles that had referenced, or were related to, articles included in this review or other relevant articles (i.e., articles that cover the topic of interest but did not fit inclusion criteria for this review), respectively. Third, reference lists of articles selected for this review, other relevant articles, and previous reviews [27, 33] were searched manually. Fourth, a search for related papers using Connected Papers [34] was conducted for articles selected for the review. Fifth, internet searches of gray literature were undertaken together with targeted searches on government websites of key policies including Australian National Disability Insurance Agency [35], United Kingdom Department of Health and Social Care [36], Government of Canada Justic Laws Website [37, 38], and the USA’s Library of Congress [39]. Sixth, targeted searches on scientific databases (e.g., SCOPUS, google scholar) for empirical studies that were identified in the gray literature reference lists, were undertaken.

### Inclusion and exclusion criteria

An article was included in this review if: (1) it empirically examined at least one mental health service (e.g., inpatient, outpatient, community service) or factors relating to the provision of mental health services (e.g., availability of psychiatrists); (2) it empirically examined at least one policy lever (i.e., tool/instrument that could be applied by a government to influence service access and/or utilization); (3) it was focused on children aged 0–17 years and 11 months with a diagnosis of autism (samples including adults were considered if the results pertaining to children were presented separately) or their parents/caregivers; and (4) it was available in an English language, peer-reviewed journal. Articles were excluded if they were (1) not available in English; (2) were not data based (e.g., books, theoretical papers, reviews); and (3) were unpublished dissertations/theses. In terms of gray literature, published government reports on policy levers were included.

### Quality assessment

The Mixed Methods Appraisal Tool (MMAT) [40] was utilized by two reviewers to evaluate study quality. The MMAT employs five criteria for each study type to assess the quality of the study. For example, the quality of quantitative non-randomized controlled trials is based on the following factors: (1) representative target population, (2) appropriateness of measures, (3) completeness of outcome data, (4) accounting for confounders, and (5) adherence to intervention administration. Reviewers had to indicate “yes,” “no,” or “can’t tell” for each criterion, with “can’t tell” indicating there was insufficient information provided to reach a decision. Two independent reviewers appraised all included studies. A third reviewer was available in case disagreements could not be resolved. The MMAT does not produce an overall quality score. An agreement on study quality was reached through discussions between the two reviewers (see Table 2 for results).

**Table 2:** Quality assessment using the mixed methods appraisal tool (2018)

Citation	Quantitative nonrandomized				
	Are the participants representative of the target population?	Are measurements appropriate regarding both the outcome and intervention (or exposure)?	Are there complete outcome data?	Are the confounders accounted for in the design and analysis?	During the study period, is the intervention administered (or exposure occurred) as intended?
Bilaver and Jordan (2013) [41]	Yes	Yes	Yes	Yes	Yes
Chatterji et al. (2015) [29]	Yes	Yes	Yes	Yes	Yes
Cidav et al. (2014) [42]	Yes	Yes	Yes	Yes	Yes
McBain et al. (2020)(43)	Yes	Yes	Yes	Yes	Yes
Stuart et al. (2017) [44]	Yes	Yes	Yes	Yes	Yes

	Quantitative descriptive				
	Is the sampling strategy relevant to address the research question?	Is the sample representative of the target population?	Are the measurements appropriate?	Is the risk of nonresponse bias low?	Is the statistical analysis appropriate to answer the research question?
Miller et al. (2016) [45]	Yes	Yes	Yes	Yes	Yes

All studies met MMAT screening questions criteria S1, “Are there clear research questions?”; and S2, “Do the collected data allow to address the research questions?”. The ‘Can’t tell’ response category means that the paper do not report appropriate information to answer ‘Yes’ or ‘No’

## Results

**Fig. 1:**
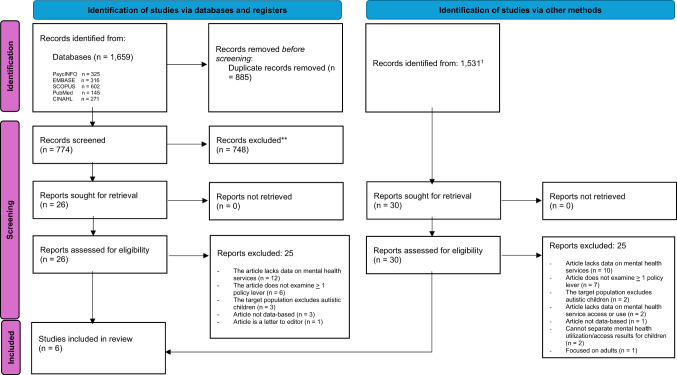
PRISMA flow diagram. ^1^Only articles from reference list and “cited by” searches on Google Scholar are reported, as these options show the total number of available articles. The total number of articles from “related articles,” Connected Papers, or grey literature searches could not be determined, thus the reported number may be an underestimation

Figure 1 presents an overview of our search strategy, and the number of articles identified and included at each stage. Initial database searches resulted in the identification of 1,659 articles (PsychInfo: 325, Embase: 316, SCOPUS: 602, PubMed:145, CINAHL: 271). Once duplicates were identified (using EndNote’s “find Duplicates” option) and removed (i.e., moved to a separate folder), a total of 774 underwent title and abstract screening. Most were excluded during title and abstract screening, with 26 articles for full text review. An additional 30 articles were found from additional sources (i.e., reference lists, cited by searches), resulting in a total of 56 articles that underwent full text review. Of these, six met inclusion criteria and were included in the review (see Fig. 1 for an overview of the search strategy and number of articles identified at each stage, see Supplementary Table 2 for a list of excluded articles). All stages of the screening and review process were undertaken by two reviewers. Disagreements pertaining to study selection and quality assessments were discussed and resolved via a consensus approach. For the title and abstract screening and full-text review, inter-rater reliability was 96% and 87.5%, respectively. As per MMAT guidelines, an overall quality score was not calculated and thus the inter-rater reliability of the quality assessment was not calculated. The quality assessment indicated that all articles were of sound methodological quality (see Table 2).

### Overview of included studies (study characteristics)

Information about the included studies is presented in Table 3. While the scope of the review did not restrict on the basis of country, the six included studies were all undertaken in the USA and published between 2013 and 2020. Of these, five were retrospective studies [29, 41–44] and one was a cross-sectional study [45]. Three studies did not specify a mental health service type [29, 41, 45], two studies focused on inpatient and outpatient services [42, 44], and one study evaluated mental health service providers [43].

**Table 3:** Overview of studies included in review

Citation	Country	Study type	Study sample	Service/service setting	Policy lever description		
						National policy lever	State Policy lever
Bilaver and Jordan. (2013) [41]	USA	Retrospective	*N* = 949 children, age range 3–17 years, male = 82%; Hispanic = 9% Hispanic, Nonwhite = 11% white = 80%. Strict condition specific (ASD) parity state: *n* = 342 Strict non–ASD specific parity state: *n* = 194 Not a strict parity state: *n* = 413	Unclear, mental health setting	State Mental Health Parity Law	-	x
Chatterji et al. (2015)[29]	USA	Retrospective	*N* = 21,158; Target group: *n* = 17,743, Comparison group: *n* = 34,15; Age range: 2–17 years, *M* age = 11.5 years; Gender and race/ethnicity distribution not reported	Unclear, mental health setting	State health insurance mandates requiring private insurance policies to cover services for autism	-	x
Cidav et al. (2014) [42]	USA	Retrospective	*N* = 47,536 Target group: *n* = 10,693 children, *M* age = 11 years, male = 80%; Black = 11%, Hispanic = 9%, Other = 17%, White = 63% Comparison group (Disability): *n* = 34,424 children, M age = 10 years, male = 79%; Black = 19%, Hispanic = 7%, Other = 26%, White = 48% Comparison group (Inpatient/Long-term care): *n* = 2,419 children, M age = 13 years, male = 81%; Black = 14%, Hispanic = 5%, Other = 19%, White = 62%	Inpatient, outpatient	1915(c) home- and community-based waivers	x	-
McBain et al. (2020) [43]	USA	Retrospective	*N* = 50 USA states and the District of Columbia; Age, gender and race/ethnicity distribution not reported	Unclear, Mental health providers	State health insurance mandates requiring private insurance policies to cover services for autism		x
Miller et al. (2016) [45]	USA	Cross-sectional	*N* = 41 states and the District of Columbia; Age, gender and race/ethnicity distribution not reported	Unclear, mental health setting	Autism-Specific 1915(c) Waiver for Children and Youth with Autism Spectrum Disorder (ASD)	x	-
Stuart et al. (2017) [44]	USA	Retrospective	*N* = 38,928 children, age range = 0–18 years. Pre-parity: *n* = 20,155, male 83% Post-parity: *n* = 28,009, male 82%. Race/ethnicity distribution is not reported	Inpatient, outpatient	Federal Parity Law: The Paul Wellstone and Pete Domenici Mental Health Parity and Addiction Equity Act (MHPAEA) of 2008	x	-

Citation	Policy lever target	Utilization	Access				
			Affordability	Accessibility	Accommodation	Availability	Acceptability
Bilaver and Jordan. (2013) [41]	Insurance carriers	x	x	–	–	–	–
Chatterji et al. (2015)[29]	Insurance carriers	–	x	–	–	x	–
Cidav et al. (2014) [42]	Insurance carriers	x	x	–	–	–	–
McBain et al. (2020) [43]	Insurance carriers	–	–	–	–	x	–
Miller et al. (2016) [45]	Insurance carriers	–	–	–	–	x	–
Stuart et al. (2017) [44]	Insurance carriers	x	x	–	–	–	–

*USA* United States of America, *x* included in study, – not included in study

Three studies focused on national policy levers [42, 44, 45], while three focused on state policy levers [29, 41, 43]. All studies were regulatory policy levers, focused on insurance companies. Two studies evaluated the 1915(c) home and community based waivers [42, 45], two looked at state insurance mandates [29, 43], and two focused on parity laws [41, 44]. Three studies evaluated utilization [41, 42, 44] and all studies examined access [29, 41–45].

### Impacts of these policy levers on mental health service utilization and access

#### Utilization

The three papers that reported on health service utilization (i.e., the use of health services [46]) were retrospective studies, two evaluated mental health parity laws [41, 44] and the other one evaluated the 1915(c) home- and community-based waivers [42].

Of the two studies examining mental health parity laws, one focused on federal laws [44] and the second centered on strict state laws [41], but neither observed a significant increase in service utilization resulting from the implementation of these laws. Using the Truven Health MarketScan Database from 2007 to 2012, Stuart, McGinty [44] evaluated the service utilization (mental health and non-mental health services; inpatient, outpatient services) of 38,928 Autistic children aged 0–18 years following the implementation of federal mental health parity laws. A non-significant trend of an increase in service utilization following the introduction of these laws was reported. Drawing on data from the National Survey of Children with Special Health Care Needs 2005–2006, Bilaver and Jordan [41] evaluated the impact of strict state mental health parity laws on mental health service utilization of 949 privately insured Autistic children aged 3–17 years. No significant difference was found between mental health service utilization for children living in (1) states with strict mental health parity laws that explicitly or implicitly covered autism (*n* = 342), (2) states with strict mental health parity laws that did not explicitly or implicitly cover autism (*n* = 194), and (3) states with no strict parity laws (i.e., states that relied on federal parity laws to set and enforce minimum standards) (*n* = 413).

Using the 2005 Medicaid Analytic Extract data files, Cidav, Marcus [42] examined the impact of 1915(c) home- and community-based waivers on service utilization for Autistic children who were waiver participants (*n* = 10,693) compared to children eligible for Medicaid through disability (*n* = 34,424) and children who had one inpatient/long-term care episode (*n* = 2,419). Autistic children (aged 11 years, mental health care claims paid under fee-for-service) who were recipients of the waiver program were significantly less likely to be hospitalized or placed in long-term care, and more likely to receive outpatient services, compared to control groups.

Taken together, these findings indicate that there is not significant evidence showing that the federal mental health parity laws increased mental health services utilization by Autistic children or their families [41, 44]. The evidence, though limited, does suggest that the 1915(c) home- and community-based waivers appear to be reducing hospitalization/institutionalization and increasing outpatient services for this population [42]. A strength of this body of research is the use of large datasets thereby reducing selection bias and improving generalizability of the findings. However, findings are limited by the fact that the data were collected over a decade ago, thus the impact of these policy levers on service utilization by the current population of Autistic children and their families is unknown.

#### Access

Drawing on the Penchansky and Thomas [47] framework, access was evaluated in regard to five dimensions, including: (1) affordability, referring to the cost of services and the individual’s ability to pay; (2) accessibility, relating to the supply and location of services, including an individual’s ability to travel to the service and available transportation; (3) accommodation, indicating the way in which services are organized to receive clients, such as walk-in facilities and telephone services; (4) availability, referring to the type and number of services; and, (5) acceptability, relating to an individual’s perception of the service. Of note, is that while acceptability is conceptualized as its own dimension for analysis purposes, it is important to acknowledge that people’s perceptions regarding service acceptability are likely shaped by the preceding four dimensions.

In our sample, four retrospective studies assessed affordability [29, 41, 42, 44] and three examined availability [29, 43, 45]. No studies evaluated accessibility, accommodation, or acceptability.

#### Affordability

Two studies evaluated mental health parity laws [41, 44], one the 1915(c) home- and community-based waivers [42], and the other the state health insurance mandates pertaining to private insurance policies regarding covering autism [29]. Stuart, McGinty [44] found increases in service utilization following the introduction of federal mental health parity laws that were not associated with an increase in out-of-pocket spending, suggesting that there was a reduction in financial burden for families. However, Bilaver and Jordan [41] did not observe a significant reduction in financial burden for families living in (1) states with strict mental health parity laws that explicitly or implicitly covered autism or (2) states with strict mental health parity laws that did not explicitly or implicitly cover autism when compared to states with no strict parity laws. Rather, they found that families with Autistic children who needed mental health services living in a strict parity law state were 61% more likely to report out-of-pocket spending greater than $1,000. Furthermore, families living in strict parity states that did specify autism, compared to those that did not specify autism, were 91% more likely to report unreasonable out of pocket spending. The differences in results between the two studies could be due to several factors, including variations in sample size and the use of different datasets across different years. Cidav, Marcus [42] found that there is a point at which the 1915(c) home- and community-based waiver program became more cost-efficient than the comparison care options. However, no state spent enough for the cost-savings to be realized. Using data from the 2005 to 2006 and the 2009 to 2010 waves of the National Survey of Children with Special Health Care Needs, Chatterji, Decker [29] evaluated the impact of state health insurance mandates on the financial burden of families with Autistic children and psychiatric disorders compared with families with Autistic children and physical health disorders. Their results found no significant association between state mandates and family’s out-of-pocket costs or perceived financial burden.

#### Availability

Of the three studies that looked at availability of services, two evaluated state insurance mandates requiring private insurance to cover services for autism [29, 43] and one looked at the autism specific 1915(c) home- and community-based waiver [45]. The autism specific 1915(c) home- and community-based waiver is an adaption of the 1915(c) home- and community-based waiver that focuses specifically on services for those with autism rather than the broader population of individuals with developmental, intellectual, and physical disabilities. Chatterji, Decker [29] reported no association between mandates and access to mental health services for privately insured families with Autistic children. McBain, Cantor [43] evaluated whether mandates led to increases in health service providers, including child psychiatrists, across 50 USA states and the District of Columbia. A significant relationship between the introduction of mandates and the number of available child psychiatrists per 100,000 children, with an increase of 0.45 and 0.53 child psychiatrists per 100,000 children in 2013 and 2017, respectively was reported. Miller, Merryman [45] evaluated the use of the autism specific 1915(c) home- and community-based waiver and the services provided under this waiver in 41 states and the District of Columbia and reported that only one state provided mental health services under this waiver.

Together, the available studies suggest that the majority of policy levers identified have not resulted in financial benefit for families of Autistic children, however, the variance in studies focused on availability makes it difficult to draw reliable conclusions. The results suggest that state insurance mandates seem to have resulted in significant increases in the supply of child psychiatrists across the USA [43]. However, there has been no association found between mandates and families accessing services [29], and these services do not appear to be provided under the autism specific 1915(c) home- and community-based waiver [45]. As mentioned above, the benefit of these studies is their use of large datasets, however, most results are outdated with new research needed to draw conclusions about the impact of these policy levers on service access for the current population of Autistic children and their families.

#### Gray literature

Although all the identified empirical studies were undertaken in the USA, gray literature searches reveal that several countries have implemented autism specific policy levers. These included Canada (Federal Framework on Autism Spectrum Disorder Act 2023; [37]), the United Kingdom (Autism Act 2009 [48] and National Strategy for Autistic Children, Young People, and Adults (2021–2026) [49]), France (Fourth National Autism Strategy (2018–2022) [50]), and the USA (Autism CARES Act [51]). While other countries such as Australia (National Disability Insurance Scheme (22)), Sweden (Swedish Disability Policy [52]), India (The Rights of Persons with Disabilities Act (2016) [53]), and Japan (Act on the Support for Persons with Developmental Disabilities (2004) [54]) had policy levers targeted at individuals with disabilities, which included autism.

Of the autism specific policies, there was evidence of regulatory (Canada’s Federal Framework on Autism Spectrum Disorder Act 2023; [37] and United Kingdom’s Autism Act 2009 [48]) organizational (National Strategy for Autistic Children, Young People, and Adults (2021–2026) [49]), and behavioral (National Strategy for Autistic Children, Young People, and Adults (2021–2026) [49]) policy levers. With the exception of the Autism Act 2009, the autism specific policies were enacted within the last six years.

Of the autism specific policies, we were only able to locate one report which assessed the impact of the United Kingdom’s Autism Act 2009 on utilization or access to mental health services by Autistic children and their families [55]. The report sought to evaluate Autistic people and their families’ views on health, education, Social care, and employment opportunities for Autistic people. Regarding mental health, the report found that only 5% of participants reported that mental health professionals were effective at supporting them; many reported that services needed to be improved by providing more consistent, long-term, better funded and tailored services; and that better access was needed to Child and Adolescent Mental Health Services. These findings were used to inform United Kingdom’s National Strategy for Autistic Children, Young People, and Adults (2021–2026) [49].

## Discussion

This systematic review synthesized the available literature evaluating the impact of government policy levers on mental health service access by Autistic children and their families. Though gray literature results indicated that governments internationally have implemented autism specific policies, only six empirical studies and one government report were identified that evaluated the impact of policy levers on mental health service utilization and access by Autistic children and/or their families. All studies were from the USA, were primarily retrospective, and centered around national and state regulatory policy levers focused on insurance companies. The government report was from the Unite Kingdom, and it evaluated the Autism Act 2009. This finding is consistent with a previous review which also found a minimal number of studies looking at the impact of policy levers on mental health service utilization or access for Autistic children and their families [27]. Due to the limited number of studies and reports identified, this review was unable to establish whether government policy levers improved mental health service utilization or access for Autistic children and their families.

For studies in this systematic review that evaluated service utilization, there was no significant evidence to suggest that federal mental health parity laws increased mental health services utilization by Autistic children or their families [41, 44], however, there was evidence showing that the 1915(c) home- and community-based waivers are having an impact on reducing hospitalization/institutionalization and increasing outpatient services for this population. In regard to the Stuart, McGinty [44] study on the The Paul Wellstone and Pete Domenici Mental Health Parity and Addiction Equity Act (2008), a trend toward significance was observed. Given that the law was enacted in 2008 and the data collected between 2007 and 2012, it is possible that not enough time had passed for a significant impact to be observed. However, subsequent non-autism focused literature has shown that since its enactment, the law has had minimal impact on improving access to, and reducing cost of, care [56]. Non-compliance by insurers and lack of enforcement by government agencies are reasons that have been put forward for the absence of impact [56]. Presskreischer, Barry [56], who evaluated state factors that affect law enforcement variation, found that implementation was impacted by several factors including relationships between insurance commissioners’ offices and other state agencies and the complexity of the laws. They argued that improving coordination among stakeholders could lead to better enforcement of the law. Given the small number of studies on the impact of federal parity laws on Autistic children and their families together with the fact that a considerable amount of time has passed since the laws introduction, more research focusing on Autistic children’s utilization of mental health services is warranted.

The studies in this systematic review that looked at service access focused primarily on service affordability [29, 41, 42, 44] and service availability [29, 43, 45], with most not finding significant improvement in affordability [29, 41, 42] or availability [29, 45] resulting from the introduction of government policy levers. In fact, the introduction of some policy levers appeared to increase a family’s financial burden [41], although the variance in studies focused on availability makes it difficult to draw conclusions. The results also suggest that state insurance mandates were correlated with significant increases in the supply of child psychiatrists across the USA [43]. However, there has been no association found between mandates and families accessing services [29], and these services do not appear to be provided under the autism specific 1915(c) home- and community-based waiver [45]. Furthermore, while McBain, Cantor [43] observed an increase in child psychiatrists following the introduction of state insurance mandates, it is unclear whether this increase led to a rise in Autistic children accessing psychiatry services and/or whether these psychiatrists had experience working with Autistic children. In addition, the observational nature of the study makes it impossible to conclude whether the introduction of state insurance mandates resulted in the increase in child psychiatrists or whether this increase was due other factors, such as the broader political climate. Given the variation in studies and that most results are dated, new research , specifically longitudinal research, focusing on more recent datasets is required to draw conclusions about the long term impact of policy levers on service access for the current population of Autistic children and their families [41].

The finding that no empirical evidence regarding the accessibility, accommodation, or acceptability of services following the introduction of policy levels is available suggests that more research targeting these areas is necessary. Neglecting these dimensions of access leads to an unclear picture of Autistic children and their family’s access to mental health services. Without research evaluating these domains, drawing definitive conclusions regarding service access is not possible.

Given the rise in youth mental health difficulties [57], the gap between need and the proportion of Autistic children receiving mental health services [12, 13], and the high percentage of Autistic children experiencing co-occurring psychiatric conditions [5], it was surprising that though globally governments have implemented policies aimed at improving service access for Autistic individuals [20, 21, 35, 36], evaluation of the impact of these policies has been neglected. While surprising, it was not unexpected given the evidence indicating that Autistic children are often excluded from research, either directly or indirectly when researchers do no segregate data based on diagnostic status [31], as well as the challenges undertaking research on policy levers, including policies rarely operating in isolation and jurisdiction differences in implementing policies [41]. Therefore, while gray literature such as the evaluation of the United Kingdom’s Autism Act 2009 provides some insight into perceptions of mental health services and their accessibility, the impact of government policy levers globally on mental health service access by Autistic children and their families remains largely unknown. This is problematic given that improving mental health service access requires understanding of which policy levers lead to successful utilization and access, and which do not. This review exposed this gap in the evidence base and draws attention to the great need for research to be undertaken evaluating the impact of policy levers on mental health service utilization and access by Autistic children and their families.

Strengths of this review included the use of multiple search strategies as well as a rigorous framework for selecting and analyzing studies. Two authors independently undertook the article selection and analysis process and achieved high inter-rater agreement. This review, however, is not without its limitations. First, the decision to include English language studies only increases the chance of bias and limits the generalizability of findings. Second, the generalizability of findings is also impacted by the small number of studies identified and that all included studies were undertaken in the USA. Third, the included studies were limited by their lack of methodological diversity (i.e., primarily retrospective studies). Fourth, while most of the included studies have been published within the last decade, the databases used are over a decade old. More current research is therefore needed to determine the impact that government policy levers are having on the current population of Autistic children and their families. Fifth, the focus on government policy levers rather than government policies may have reduced the number of articles identified and included. The decision was made to focus on government policy levers, rather than government policies which are often broad in nature, to be in keeping with the five categories outlined by Roberts, Hsiao [19]. This structure provides a framework for cross-context comparisons. Sixth, 50% [29, 43, 45] of studies did not report gender distribution while 66% [29, 43–45] did not report race/ethnicity distribution. Furthermore, when gender was reported, over 80% of the samples were male. The limited sociodemographic information and primarily male samples, limit the applicability of these findings.

To conclude, this review found that while governments internationally have implemented autism specific policy levers, few studies have been undertaken to evaluate the impact of these levers on mental health service utilization and access by Autistic children and their families. Though limited, the available evidence from the USA suggests most policy levers have not resulted in greater utilization or access of mental health services for Autistic children with mental health difficulties and their families. This review highlights the need for more evidence, especially given that this information would be instrumental in informing government decisions regarding service organization and funding allocation.

## Data Availability

No datasets were generated or analysed during the current study.
